# Quantitative trait locus mapping for important yield traits of a sorghum-sudangrass hybrid using a high-density single nucleotide polymorphism map

**DOI:** 10.3389/fpls.2022.1098605

**Published:** 2022-12-20

**Authors:** Qianqian Lu, Xiaoxia Yu, Huiting Wang, Zhuo Yu, Xia Zhang, Yaqi Zhao

**Affiliations:** Agricultural College, Inner Mongolia Agricultural University, Hohhot, Inner Mongolia, China

**Keywords:** sorghum-sudangrass hybrid, ultra-high-density genetic map, F2 population, yield traits, quantitative trait locus mapping, candidate gene prediction

## Abstract

The sorghum-sudangrass hybrid is a vital gramineous herbage.The F2 population was obtained to clarify genetic regularities among the traits of sorghum-sudangrass hybrids by bagging and selfing in the F1 generation using ‘scattered ear sorghum’ and ‘red hull sudangrass.’ This hybrid combines the characteristics of the strong resistance of parents, high yield, and good palatability and has clear heterosis. A thorough understanding of the genetic mechanisms of yield traits in sorghum-sudangrass hybrids is essential in improving their yield. Therefore, we conducted quantitative trait locus (QTL) mapping for plant height, stem diameter, tiller number, leaf number, leaf length, leaf width, and fresh weight of each plant in three different environments, using a high-density genetic linkage map based on single nucleotide polymorphism markers previously constructed by our team. A total of 55 QTLs were detected, uniformly distributed over the 10 linkage groups (LGs), with logarithm of odds values ranging between 2.5 and 7.1, which could explain the 4.9–52.44% phenotypic variation. Furthermore, 17 yield-related relatively high-frequency QTL (RHF-QTL) loci were repeatedly detected in at least two environments, with an explanatory phenotypic variation of 4.9–30.97%. No RHF-QTLs were associated with the tiller number. The genes within the confidence interval of RHF-QTL were annotated, and seven candidate genes related to yield traits were screened. Three QTL sites overlapping or adjacent to previous studies were detected by comparative analysis. We also found that QTL was enriched and that qLL-10-1 and qFW-10-4 were located at the same location of 25.81 cM on LG10. The results of this study provide a foundation for QTL fine mapping, candidate gene cloning, and molecular marker-assisted breeding of sorghum-sudangrass hybrids.

## 1 Introduction

The sorghum-sudangrass hybrid (*Sorghum bicolor* × *Sorghum sudanense* [Piper] Stapf) is an annual warm-season forage that provides water and soil conservation benefits. It combines the advantages of high grass and large leaf yield, improved tillering, good regeneration, and high nutritional value from both parents, providing substantial heterosis. Cattle, sheep, geese, ducks, poultry, and other livestock utilize this hybrid as feed. The sorghum-sudangrass hybrid presents a developed root system, good soil capacity, and strong ecological adaptability and further presents broad development and utilization prospects in environmental protection and animal husbandry, especially the development of the breeding industry in agricultural areas (Saballos et al., 2008; Han et al., 2015; Mahmoudzadeh and Oad, 2018; Peng et al., 2020). In recent years, with the rapid development of animal husbandry and frequent extreme weather worldwide, increasing demand for high-quality forage has indicated a shortage of high-quality green and fresh forage. This is the main reason for limiting the quality and efficiency of animal husbandry in the northwestern Himalayan region of India (Mir et al., 2020; Kumar et al., 2022), the southern part of the Korean Peninsula and Jeju Island (Peng et al., 2020), the northern and southeastern United States (Wells et al., 2014; Nave et al., 2020), northern Europe (Liatukienė et al., 2008), Canada (Bélanger et al., 2006), and parts of China (Fang, 2018). Therefore, increasing forage yield is crucial in meeting sufficient livestock nutrition and accelerating production. Recently, the sorghum-sudangrass hybrid has been widely cultivated and applied due to its high grass yield, strong resistance, rich nutrient content, and wide adaptability, which will ultimately play a positive role in sustainable animal husbandry development and alleviate the contradiction between supply and demand of herbage.

Forage grass is a crucial material base of animal husbandry and the main source of livestock nutrition (Li et al., 2015; Zhang et al., 2021). Plant height (PH), stem thickness (ST), tiller number (TN), leaf-related traits (leaf number (LN), leaf length (LL), and leaf width (LW), and fresh weight (FW) represent the most important yield traits. An understanding of the genetic basis of these traits carries considerable importance regarding increasing the output of sorghum-sudangrass hybrids (Ma et al., 2018). In the past decades, gene identification related to crop target traits was mainly achieved *via* hybrid population breeding (Girma et al., 2019). Researchers from the United States, India, Japan, Australia, and China have cultivated several forage varieties, such as Jianlibao (Jumbo), Leyi (Everlush), Mengong Green series forage, Wancao No. 2 (WC-2), and Jicao No. 1 (JC-1) (Han et al., 2015). However, conventional breeding results from the perennial selection of crop hybrid populations for important phenotypic traits, which are time-consuming and inefficient regarding targeted modifications for complex traits (Huang and Yan, 2019; Islam et al., 2021). With molecular biology’s rapid development, molecular marker-assisted breeding has received increasing attention in forage breeding. Understanding the quantitative trait locus (QTL) function associated with important yield traits is an effective way to improve crop yield.

QTL mapping has been widely used to study yield traits in sorghum-sudangrass hybrids. Shi et al. (2017) constructed genetic linkage maps based on amplified fragment length polymorphism (AFLP) molecular markers and detected 13, 11, 10, 2, and 9 QTLs for stem diameter (SD), LL, TN, LW, and LN, respectively. Wang et al. (2021) detected four QTLs associated with PH and one QTL related to TN based on a genetic map containing 133 SSR markers. Using a genetic linkage map containing 181 SSR markers, Yu et al. (2018) further detected two QTLs related to LN, LL, LW, and TN each.; four QTLs with SD; and one QTL associated with PH. Liu et al. (2015) constructed a genetic linkage map with 124 SSR markers and identified one QTL each for PH, SD and TN, and three QTLs each for LN and FW. However, genetic maps constructed using traditional molecular markers, such as simple-sequence repeats (SSR) and AFLP, have low density and few QTL sites, which complicates meeting the needs of further QTL fine mapping (West et al., 2006).

With the gradual increase in available plant genome sequences, the combination of next-generation sequencing technology and reference genomes provides a new research strategy for QTL mapping and molecular marker-assisted breeding of important plant traits (Hart et al., 2001). The genome size of sorghum is about 730 Mb. Since the sudangrass and sorghum-sudangrass hybrid genome sequencing was incomplete, we used the sorghum genome as a reference genome to develop single nucleotide polymorphism (SNP) markers for sorghum-sudangrass hybrid. Compared to AFLP and SSR markers, SNP markers are abundant and polymorphic, allowing high-density genetic map construction (Wen et al., 2020). A single genetic linkage map has been constructed using SNP markers in sorghum-sudangrass hybrids (Jin et al., 2021). SNP markers can be developed on a large scale through different high-throughput sequencing technologies, such as genotyping-by-sequencing (GBS) (Elshire et al., 2011), restriction site-related DNA tag sequencing (RAD-seq) (Xu et al., 2018), specific-length amplified fragment sequencing (SLAF-seq) (Sun et al., 2013), and whole-genome resequencing (WGRS) (Li et al., 2009). WGRS is a sequence-based genotyping method that is not limited by a restriction site and can quickly obtain a considerable number of recombinant breakpoints and marker density. According to research needs, sequencing coverage can be adjusted to the whole or part of the population, which vastly improves the accuracy of QTL mapping (Hart et al., 2001). WGRS has been successfully applied to map construction and QTL mapping of related traits of a variety of important crops, such as rice (Smulders et al., 2019), sorghum (Zhang et al., 2021), and peanuts (Jiang et al., 2021). However, WGRS has not been used in QTL mapping for traits related to the sorghum-sudangrass hybrid, and it is rarely reported in gene prediction.

Therefore, the aim of this study was to conduct QTL mapping analysis for seven traits (i.e., PH, SD, TN, FW, and the leaf-related traits LN, LL, and LW), and the genes in the stably detected QTL intervals were annotated based on the high-density SNP map of sorghum-sudangrass hybrids constructed in previous research by our group. These results will serve as a foundation for further studies such as QTL fine mapping, functional key gene verification, and marker-assisted breeding.

## 2 Materials and methods

### 2.1 Plant material

**Figure 1:**
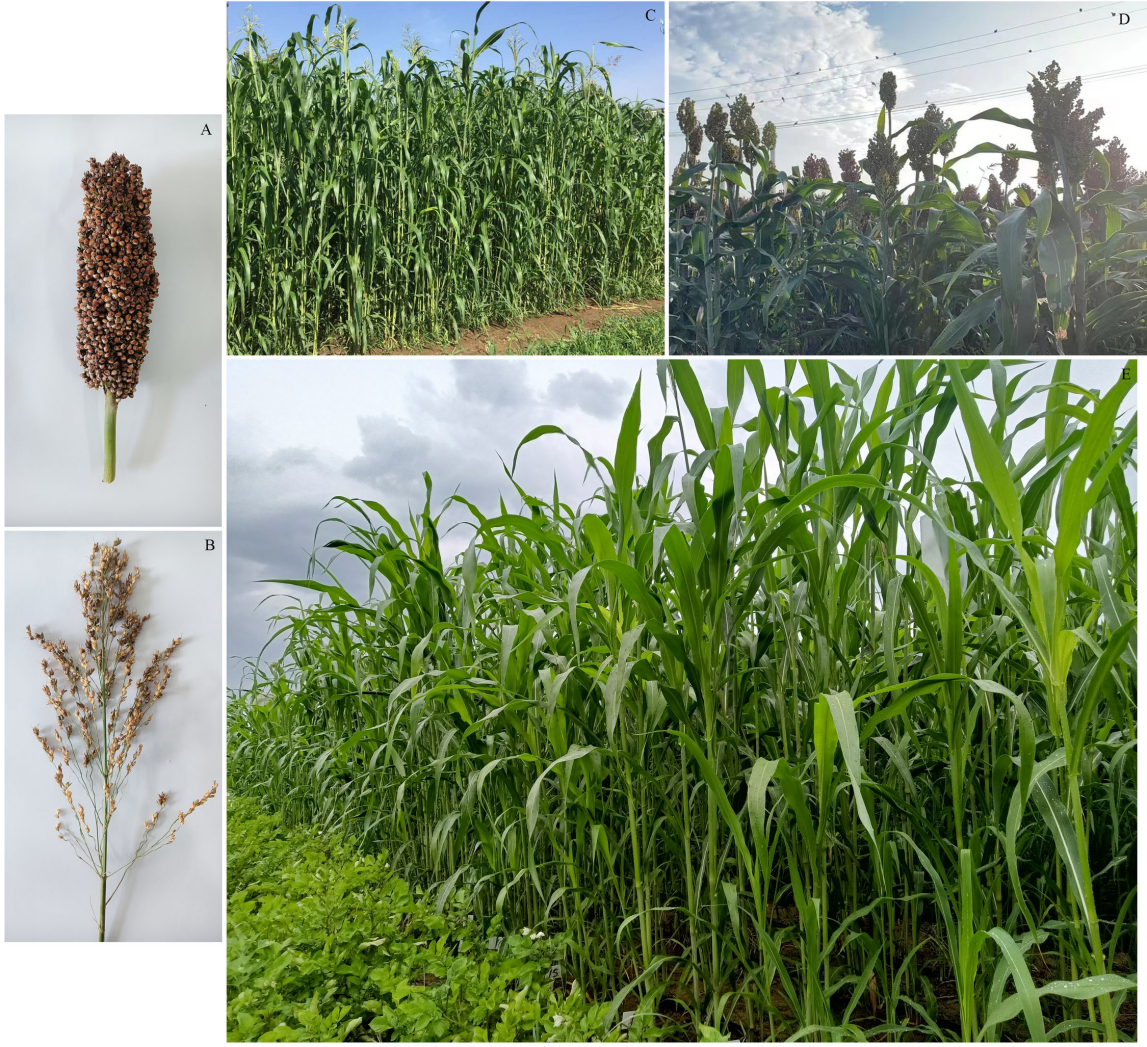
Phenotype and field growth of parents and part of F2 population of sorghum-sudangrass hybrid. **(A)** Scattered ear sorghum (♀); **(B)** Red hull sudangrass (♂); **(C)** Male parent growing in the field; **(D)** Female parent growing in the field; **(E)** F_2_ population growing in the field.

Sorghum-sudangrass hybrid F_2_ populations and the parental material were obtained by bagging and selfing in the F_1_ generation using ‘scattered ear sorghum’ and ‘red hull sudangrass’ as parents, which were provided by the College of Agronomy, Inner Mongolia Agricultural University. Each was separated by their tillers per seedlings, cloned after tissue propagation, and stored in a group culture room. The female sorghum is the main cultivar in northeast China, while the male sorghum is widely cultivated in Inner Mongolia. There are substantial differences in agronomic traits such as PH, SD, TN, FW, and leaf-related traits (LN, LL, and LW) between the two varieties (**Figure 1**).

### 2.2 Site conditions

Three field experiments were performed in Khorqin Grassland Station of Tongliao, Inner Mongolia (T), and a teaching farm in the East Zone of Inner Mongolia Ancient Agricultural University in Hohhot (H), Inner Mongolia, in 2021 and 2022. These were recorded as 2021-H, 2022-H, and 2022-T. The randomized complete block design (RCBD) was used in the field experiment and was repeated three times at each field test site. Three plants were used for each replication. The F_2_ population and parents were transplanted into the field at the end of April each year, using a plant and row spacing of 20 and 45 cm, respectively. The soil type of the test site was meadow and sandy loam soil, with moderate soil fertility and good irrigation conditions. Timely tillage, weeding, and insect and disease control management were performed throughout the growing season.

### 2.3 Trait measurement and data analysis

At the full flowering stage, each trait index of 150 F_2_ isolated individual plants and their parents were determined. The PH was measured from the plant root to the spike tip, and the LL and LW of the second leaf were measured. The SD was measured from the middle of the stem near the soil using vernier calipers, and the LN and TN of each plant were recorded. Finally, the whole plant shoot was weighed to determine the FW (Liu et al., 2015).

Excel 2010 (Microsoft Corp., Redmond, WA, USA) and SPSS 22.0 software (SPSS, Chicago, Illinois, USA) were used to calculate the phenotypic data of different traits and perform analysis of variance (ANOVA). The OriginPro software (OriginLab, Northampton, MA, USA) was used to construct the characteristics of frequency distribution and correlation analysis.

### 2.4 QTL mapping analysis

Based on SNP markers developed using WGRS, our research group previously constructed a high-density genetic linkage map of sorghum-sudangrass hybrids, which contains 5656 SNP markers distributed in 10 linkage groups (LGs), with ‘scattered ear sorghum’ and ‘red hull sudangrass’ as parents and 150 individual F_2_ isolates as the test material (Lu et al., 2022). The total length of the covered genome was 2192.84 cM, and the average distance between markers was 0.39 cM. Based on the high-density genetic linkage map, the composite interval mapping (CIM) method of WinQTL software (https://brcwebportal.cos.ncsu.edu/qtlcart/WQTLCart.htm) was used to locate and analyze the phenotypic values of PH, SD, TN, LN, LL, LW, FW and their average values (AV) in three environments (2021-H, 2022-H, and 2022-T). The Logarithm of the odds (LOD) threshold for QTL was verified by 1000 arrangements with P <0.05. The final genetic map was constructed using Mapchart 2.2 (https://www.mapchart.net). The QTL locus positions were annotated. The QTLs detected in more than two environments were defined as relatively high-frequency QTLs (RHF-QTLs) (Guo et al., 2020). QTL loci with two or more traits that overlap or were in close proximity were defined as QTL clusters, and QTLs that could explain more than 10% of phenotypic variation in different environments were considered major QTLs (Yang et al., 2022). The QTLs were named as follows: “q + trait abbreviation + linkage group + QTL serial number,” for example, in “qPH-7-1,” “q” is the QTL abbreviation, “PH” represents plant height, “7” represents the 7th linkage group, and “1” represents the first QTL on linkage group 7 (McCouch and Xiao, 1998; Ma et al., 2018).

### 2.5 Candidate gene annotation and prediction

Samples from the previous study (Lu et al., 2022) that used ‘scattered ear sorghum’ and ‘red hull sudangrass’ for high-throughput sequencing of the whole genome were re-sequenced with the Illumina HiSeqTM PE150 platform (Illumina, San Diego, CA, USA). The parental genotypes were sequenced separately at a sequencing depth of 29.71× and 28.77×, and 227.2 Mb and 234.42 Mb of data were collected, respectively. The specific steps of candidate gene prediction are as follows: (1) Detect SNP sites (different and homozygous sites) between parents, which were aligned to the reference genome sorghum *Sorghum bicolor* (sorghum) (https://phytozome-next.jgi.doe.gov/info/Sbicolor_v3_1_1) using Burrows-Wheeler Aligner (BWA) (http://bio-bwa.sourceforge.net/). Duplicate parts (rmDup) were removed using SAMTOOLS (https://www.htslib.org). The Bcftools command “merge” was used to combine ‘scattered ear sorghum’ and ‘red hull sudangrass’ genotypes, and SNP loci (different and homozygous) were retained. (2) On the basis of the results of step 1, in if the E value was ≤ 1e^-10^, BLAST (https://blast.ncbi.nlm.nih.gov/) was used to map markers on both sides of the QTL confidence interval to the physical location of the sorghum genome, to determine the variation sites in the target interval. (3) Based on the functional annotation of sorghum homologous genes, candidate genes for yield traits of sorghum were identified from the mutant loci of the second step.

## 3 Results

### 3.1 Phenotype and correlation analysis of yield-related traits

**Figure 2:**
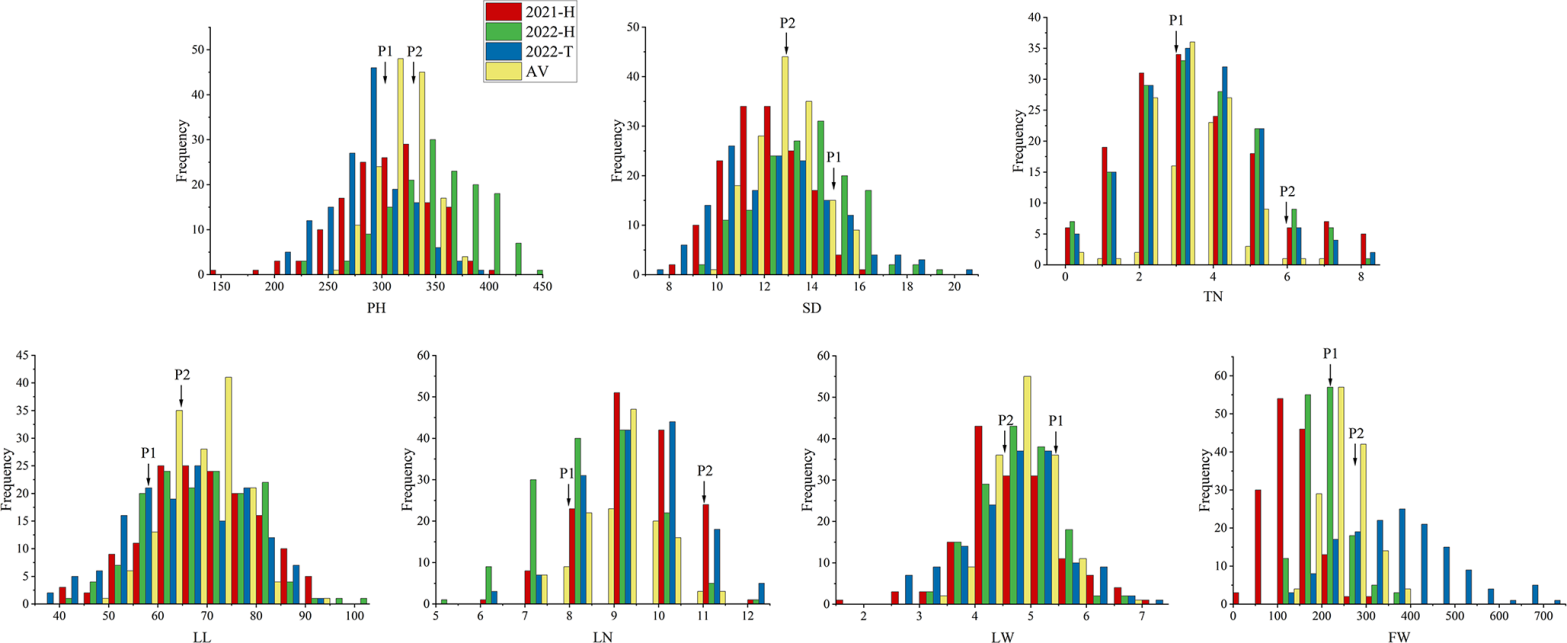
Frequency distribution maps of each yield-related trait in the F_2_ population in three environments. The traits are plant height (PH), stem diameter (SD), tiller number (TN), leaf length (LL), leaf width (LW), leaf number (LN) and fresh weight (FW). Red, green, blue, and yellow labels indicate 2021-H, 2022-H, 2022-T, and AV, respectively. 2021-H, 2022-H, 2022-T and AV represent Hohhot (2021), Hohhot (2022), Tongliao (2022) and the average environment, respectively.

Phenotypic values of seven traits of the F_2_ population and their parents (scattered ear sorghum (P1) and red hull sudangrass (P2)) are shown in **Table 1** and **Supplementary Table 1**. Each parent trait showed significant differences in different environments. The SD (AV: 14.95 vs. 12.98) and LW (AV: 5.4 vs. 4.6) of P1 were higher than those of P2, while the opposite was true for the other five traits (PH, TN, LN, LL, and FW). The genetic variation of each trait in the F_2_ population was high, and the coefficient of variation was between 7.5 and 57.74 (**Table 1**). The results of ANOVA showed that genotype had a significant influence on all traits (P <0.001 or P <0.01), and environmental factors had a significant influence on all traits except TN and LW (P <0.001) (**Table 2**). All traits had super parental separation in the 2021-H, 2022-H, and 2022-T environments and the average environment. The skewness and kurtosis of each trait were between 0.51–0.77 and 0.64–0.99, respectively. The absolute value was <1, which meets the normal distribution characteristics and is suitable for the next QTL localization study (**Figure 2**).

**Table 1:** Phenotypic analysis for seven quality traits of sorghum-sudangrass hybrids.

Trait ^a^	Environment ^b^	Parent ^c^		F_2_ population						
		P1	P2	Max	Min	AV ^d^	SD ^e^	CV(%) ^f^	Skewness	Kurtosis
PH	2021-H	227.5 ± 0.49**	340.2 ± 0.45	418	148	308.19	42.36	13.74	-0.51	0.96
	2022-H	189.6 ± 0.33**	330.8 ± 0.48	454	233	355.27	43.59	12.27	-0.36	-0.01
	2022-T	184.1 ± 0.36**	328.2 ± 0.44	395	208	285.93	35.66	12.47	0.18	0.20
	AV	200.69 ± 0.24**	333.1 ± 0.31	377	252	316.46	23.81	7.5	-0.09	-0.18
SD	2021-H	14.86 ± 0.36**	13.35 ± 0.33	16	8.16	12.11	1.59	13.12	0.01	-0.51
	2022-H	15.02 ± 0.35**	12.37 ± 0.46	19	9.02	13.51	1.92	14.21	0.02	-0.17
	2022-T	14.97 ± 0.28**	13.21 ± 0.45	20.02	7.92	12.23	2.43	19.86	0.51	-0.01
	AV	14.95 ± 0.16**	12.98 ± 0.13	15.67	9.01	12.62	1.35	10.69	0.05	-0.27
TN	2021-H	2 ± 0.33**	5 ± 0.42	8	0	3.29	1.89	57.74	0.58	-0.04
	2022-H	3 ± 0.33**	6 ± 0.31	8	0	3.32	1.74	52.40	0.23	-0.36
	2022-T	3 ± 0.33**	6 ± 0.33	8	0	3.31	1.65	49.84	0.32	0.04
	AV	3 ± 0.22**	6 ± 0.15	7	0	3.31	1.06	32.02	1.13	0.94
LN	2021-H	9 ± 0.22**	6 ± 0.25	12	6	9.34	1.14	12.21	-0.26	-0.25
	2022-H	7 ± 0.25**	12 ± 0.34	12	5	8.36	1.29	15.43	0.005	-0.27
	2022-T	8 ± 0.31**	10 ± 0.31	12	6	9.27	1.26	13.59	-0.14	-0.10
	AV	8 ± 0.15**	11 ± 0.15	11	7	8.99	0.83	9.23	-0.09	-0.10
LL	2021-H	63.4 ± 0.42**	69.5 ± 0.47	94	41.9	69.81	11.16	15.98	-0.11	-0.47
	2022-H	58.9 ± 0.52**	64.1 ± 0.32	101.6	43.5	69.54	10.83	15.57	0.13	-0.24
	2022-T	53.5 ± 0.43**	61.0 ± 0.52	91	38	65.44	11.97	18.29	-0.08	-0.64
	AV	58.6 ± 0.20**	64.9 ± 0.27	92.3	49.1	68.26	7.39	10.82	-0.09	0
LW	2021-H	5.7 ± 0.34**	4.27 ± 0.14	7.2	1.8	4.71	0.84	17.83	0.10	0.99
	2022-H	5.0 ± 0.51**	3.39 ± 0.12	6.5	3.3	4.77	0.65	13.62	0.07	-0.28
	2022-T	5.4 ± 0.27**	4.0 ± 0.16	7	2.6	4.72	0.89	18.85	-0.21	-0.14
	AV	5.4 ± 0.24**	3.9 ± 0.04	6.5	3.1	4.73	0.54	11.41	-0.10	0.49
FW	2021-H	148.8 ± 0.49**	233.3 ± 0.61	331.4	41.5	142.1	52.11	36.67	0.68	0.99
	2022-H	168.5 ± 0.35**	233.1 ± 0.64	374	102.2	211.3	48.81	23.10	0.77	0.98
	2022-T	343.9 ± 0.40**	282.9 ± 0.53	712	140	367.3	122.43	33.32	0.52	0.56
	AV	220.4 ± 0.25**	279.8 ± 0.35	392.4	140.1	240.2	52.38	21.80	0.37	-0.19

a PH, plant height; SD, stem diameter; TN, tiller number; LN, leaf number; LL, leaf length; LW, leaf width; FW, fresh weight. The units for PH, LL, LW are cm, SD is mm, FW is g.

b The populations planted in Hohhot in 2021 (2021-H); Hohhot in 2022 (2022-H); and Tongliao in 2022 (2022-T).

c P1, Scattered ear sorghum (♀); P2, Red hull sudangrass (♂).

d AV, Mean of the F_2_ population.

e SD, Standard deviation.

f CV, coefficient of variation (SD/AV*100%). ** Indicates significance at the 0.01 level.

**Table 2:** Analysis of variance (ANOVA) for seven traits in three environments.

Trait	Factor	Sum of squares	Mean square	F
PH	Environment	1128193.65	564096.83	301.55***
	Genotype	71589.12	35794.56	19.14***
	Error	2508515.41	1870.63	
SD	Environment	540.98	270.49	29.23***
	Genotype	665.81	332.91	35.97***
	Error	12410.22	9.25	
TN	Environment	0.16	0.08	0.014
	Genotype	74.62	37.31	6.53**
	Error	7660.81	5.71	
LL	Environment	5389.44	2699.22	11.21***
	Genotype	3893.14	1946.57	8.09***
	Error	322805.89	240.72	
LW	Environment	0.87	0.44	0.33
	Genotype	14.01	7.0	5.33**
	Error	1761.1	1.31	
LN	Environment	269.85	134.93	23.12***
	Genotype	92.80	46.40	7.95***
	Error	7825.97	5.84	
FW	Environment	11981607.16	5990803.58	579.85***
	Genotype	98111.79	49055.89	4.75***
	Error	13854676.43	10331.60	

*** and ** Indicates significance at the 0.001 and 0.01 level, respectively.

**Figure 3:**
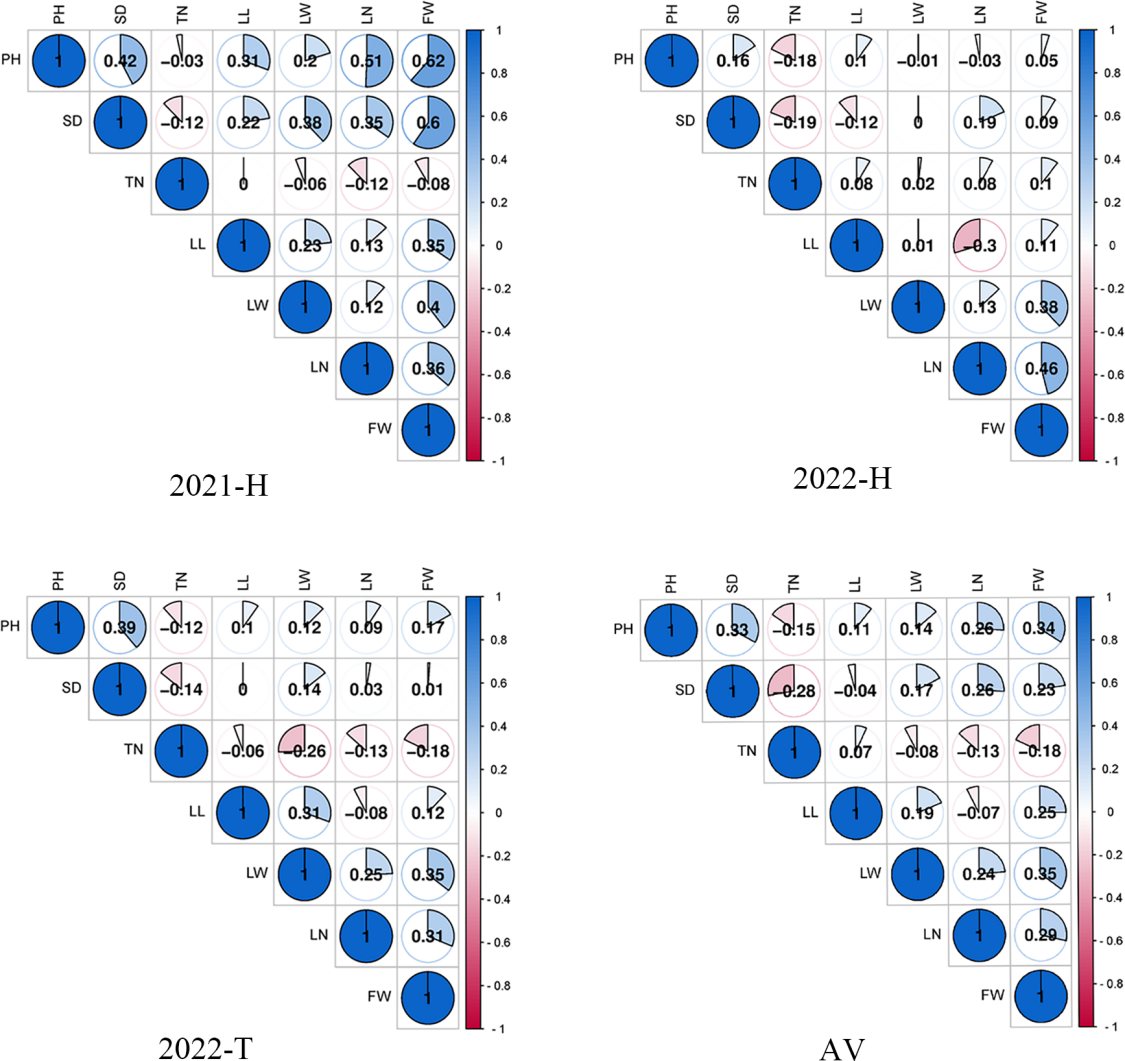
Pearson correlation coefficient (r) between traits in different environments.

Seven yield correlations were evaluated, and the results are shown in **Figure 3**. In the 2021-H, 2022-H, and 2022-T environments as well as the average environment, the Pearson correlation coefficient (r) in TN was the least significantly correlated with FW. All other traits were positively correlated with FW. In 2022-H, compared Compared with the other environments, the phenotypic difference in 2022-H changed substantially, indicating that the planting environment changed considerably. In the three environments and the average environment, the Pearson correlation coefficients (r) among leaf-related traits (LL, LW and LN) were significant, LN was significantly correlated with SD and PH, and LN was negatively correlated with SD. This was consistent with the research results of Jin et al. (2021). Consequently, we speculate that the genes controlling TN and SD traits may restrict each other. Therefore, leaf-related traits (LL, LW, and LN), FW, SD, and PH or LN may be in the same QTL cluster (i.e., with close or overlapping confidence intervals) and play a pleiotropic role in phenotypic control.

### 3.2 QTL mapping

**Figure 4:**
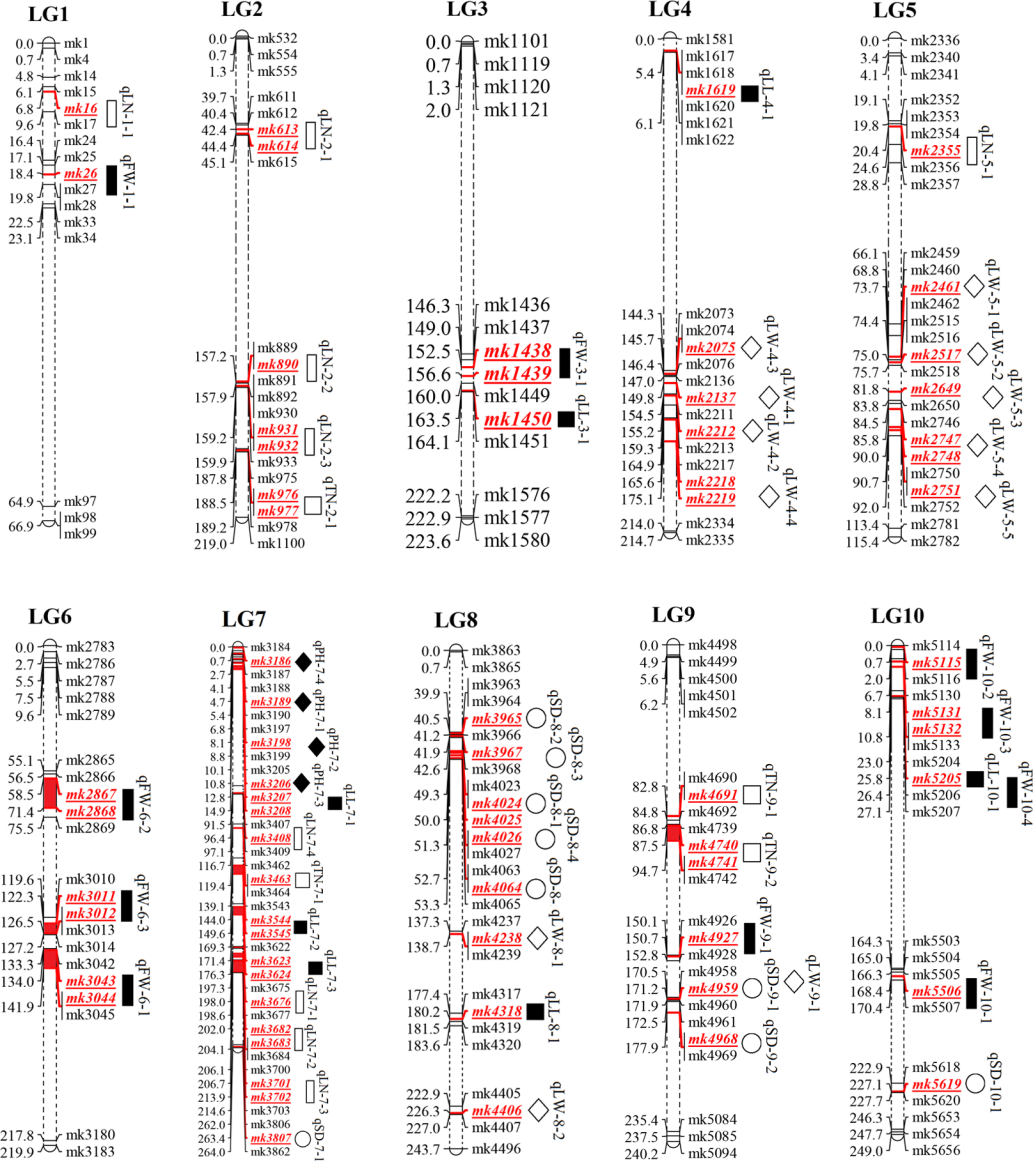
QTL mapping of yield traits in F_2_ population of sorghum-sudangrass 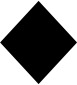 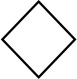 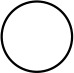 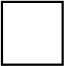 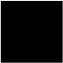 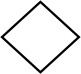 
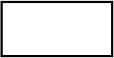
 
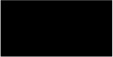
 represented QTLs for plant height (PH), stem diameter (SD), tiller number (TN), leaf length (LL), leaf width (LW), leaf number (LN) and fresh weight (FW), respectively.

The phenotypic data of PH, SD, TN, LL, LW, LN, and FW in the three environments and the average environment were analyzed by QTL mapping. A total of 266 QTLS QTLs were detected, which were distributed on 10 chromosomes, and the phenotypic variation explained ranged from 0 to 52.42% (**Supplementary Table 2**). The results revealed that 55 major QTLs and RHF-QTLs were detected, which were relatively evenly distributed in different intervals of 10 linkage groups. This included four PH-related QTLs, nine SD-related QTLs, four TN-related QTLs, and seven LL-related QTLs. There were 12 QTLs for LW, 9 for LN, and 10 for FW (**Table 3** and **Figure 4**). The LOD values ranged from 2.5–7.1, which could explain the 4.9–52.44% phenotypic variation. There were 50 major QTLs with genetic contribution >10%, and 17 RHF-QTLs were detected in at least two environments, which were relatively evenly distributed among 10 linkage groups. These traits were reported for the first time based on the high-density genetic map, which could provide the foundation for improved yield and the improvement of important agronomic traits of the sorghum-sudangrass hybrid. The specific positioning is indicated in **Table 3**.

**Table 3:** Quantitative trait loci (QTLs) identified for seven traits across three different environments (major and relatively high-frequency QTL [RHF-QTL]).

Traits ^a^	QTLs	Treatments	Chrom ^b^	Position	Marker interval	LODs ^c^	Additive effects ^d^	R^2^ (%) ^e^
PH	qPH-7-1	2021-H	7	4.71	4.4-5.3	3.3	15.94	9.2
		2022-T			4.4-5.5	5.2	16.45	10.25
		AV			4.2-5.4	3.4	9.8	11.66
	qPH-7-2	2022-H	7	8.11	7-9.4	3.1	16.00	7.76
		2022-T			7-9.4	4.4	16.73	13.56
		AV			7-10.5	3.0	9.83	12.45
	qPH-7-3	2022-T	7	10.81	10.5-11.8	4.1	14.76	18
	qPH-7-4	AV	7	0.71	0-3	3.4	8.82	13
SD	qSD-7-1	2022-T	7	263.01	261.3-263.4	3.1	-0.79	12.15
	qSD-8-1	2021-H	8	49.31	48.3-50.5	3.2	-0.59	9.7
		AV			48.7-50	4.2	-0.75	14
	qSD-8-2	2021-H	8	40.51	39.9-41.2	2.6	-0.51	9.5
		AV			40.2-41.2	3.7	-0.41	13.05
	qSD-8-3	2021-H	8	41.91	41.2-42.6	2.6	-0.47	9.04
		AV			41.2-43.1	3.4	-0.45	11.77
	qSD-8-4	AV	8	51.31	51-51.6	3.6	-0.64	15.29
	qSD-8-5	AV	8	52.71	52.5-54.4	5.0	-0.68	19.16
	qSD-9-1	2021-H	9	171.21	170.5-171.4	7.1	-0.92	25.62
	qSD-9-2	2021-H	9	177.91	177.3-178.3	3.9	-0.62	14.52
	qSD-10-1	2022-T	10	226.91	225.4-228.7	4.7	-0.48	12.19
		AV		227.71	225.9-230.2	3.2	-0.12	6.0
TN	qTN-2-1	2022-T	2	188.51	187.7-191.2	3.35	-0.62	11.15
	qTN-7-1	2022-T	7	119.41	118.5-121.8	4.1	0.74	17.56
	qTN-9-1	2022-H	9	82.81	82.2-84.8	3.4	0.68	14.7
	qTN-9-2	2022-H	9	90.51	84.8-94.5	3.1	0.78	15.86
LL	qLL-3-1	2021-H	3	163.51	162.4-163.8	3.1	-2.67	10.1
	qLL-4-1	2021-H	4	5.41	5-6.1	3.6	-2.49	10.32
	qLL-7-1	2021-H	7	13.81	12.8-14.8	3.1	6.57	10.21
	qLL-7-2	2021-H	7	148.01	144.3-150.3	3.2	-3.69	11.36
	qLL-7-3	2021-H	7	174.41	172.9-176.5	4.4	-7.03	21.65
	qLL-8-1	2022-H	8	180.21	179.1-181.6	5	2.82	17.06
	qLL-10-1	2021-H	10	25.81	24.2-26.6	4	-2.8	11.61
		AV			23-26.6	3.5	-1.41	9.11
LW	qLW-4-1	2021-H	4	149.11	145.6-151.8	3	-0.3	8.00
		2022-H			147.8-149.8	2.8	-0.19	9.83
	qLW-4-2	2021-H	4	155.21	153.2-156.4	3.2	-0.33	10.71
	qLW-4-3	2022-T	4	145.71	143.2-149.4	2.9	0.05	7.61
		AV			143.5-146.6	3.2	-0.07	8.50
	qLW-4-4	2022-T	4	170.61	168.7-173.7	3.0	-0.51	25.95
	qLW-5-1	2021-H	5	73.71	71.3-74.4	3.9	0.25	12.60
	qLW-5-2	2021-H	5	75.01	74.4-76.4	4.2	0.28	13.70
	qLW-5-3	2021-H	5	81.81	81-83.8	5.1	0.22	14.58
	qLW-5-4	2021-H	5	86.81	86.4-90	3.7	0.23	12.04
	qLW-5-5	2021-H	5	90.71	90-91.8	3.48	0.28	10.79
	qLW-8-1	AV	8	138.31	136.3-139.1	4.2	-0.16	13.48
	qLW-8-2	AV	8	226.31	225.4-227.2	4.6	-0.12	15.22
	qLW-9-1	2011-H	9	172.21	171-171.6	3.5	-0.31	10.65
LN	qLN-1-1	AV	1	6.81	6.2-8.4	3.3	0.33	14.54
	qLN-2-1	AV	2	43.41	42.4-45.1	3.1	-0.15	10.57
	qLN-2-2	AV	2	157.21	155.9-157.9	3.1	-0.16	9.39
	qLN-2-3	AV	2	159.21	157.9-159.7	3.5	-0.14	10.1
	qLN-5-1	2011-T	5	20.41	19.8-22.3	3	-0.54	14.37
	qLN-7-1	2021-H	7	198.01	197.8-198.9	4.2	-0.35	24.67
	qLN-7-2	2021-H	7	203.01	202-203.5	4.1	-0.43	25.33
		AV			203-204.1	2.6	-0.3	16.38
	qLN-7-3	2021-H	7	210.81	208.3-212.4	5.4	-0.66	30.97
		AV		208.81	204.1-211.7	2.6	-0.37	12.75
	qLN-7-4	2021-H	7	96.41	94.5-96.6	3.9	-0.58	14.84
		AV			94.4-98.4	2.6	-0.32	5.96
FW	qFW-1-1	2022-T	1	18.41	18.1-20.1	5	26.48	14.61
		AV		19.41		3.1	25.1	16.54
	qFW-3-1	AV	3	154.51	152.6-155.8	3.3	31.69	20.01
	qFW-6-1	2022-T	6	138.01	135.6-140.9	3.1	51.24	21.39
		AV		137.01	134.9-139.8	3.6	20.63	21.22
	qFW-6-2	AV	6	62.51	60.1-65.9	3.3	23.18	52.44
	qFW-6-3	AV	6	125.31	123.3-126.5	3.3	12.49	11.40
	qFW-9-1	2022-T	9	150.71	150.1-151.8	5.6	1.58	5.05
		AV			150.1-151.9	4.1	3.69	5.6
	qFW-10-1	2021-H	10	168.41	167.7-169.3	5.2	23.49	8.1
	qFW-10-2	2022-H	10	1.17	0.7-3.4	5.1	19.83	21.8
	qFW-10-3	2022-H	10	9.11	7.2-10.4	5.7	22.15	22.94
		AV			8.5-10.1	2.5	21.02	4.9
	qFW-10-4	2022-T	10	25.81	23-26.4	2.6	-41.02	8.26
		AV			23-19.6	3.4	-20.11	12.75

a The traits are PH, plant height; SD, stem diameter; TN, tiller number; LN, leaf number; LL, leaf length; LW, leaf width; FW, fresh weight 2021-H, 2022-H, 2022-T and AV represent the populations planted in Hohhot in 2021 (2021-H); Hohhot in 2022 (2022-H); Tongliao in 2022 (2022-T). and Mean of the F2 population respectively.

b Chrom, chomosome.

c LOD, logarithm of odds.

d Additive effect, positive effect was contributed by P1, negative effect was contributed by P2.

e R^2^, Phenotypic variation.

For PH, four QTLs were detected on LG7 (**Table 3** and **Figure 4**), with QTL ranging from 2.6–7.1, and the phenotypic variation rate was between 9.2% (2021-H, qPH-7-1) and 18.0% (2022-T, qPH-7-3). The qPH-7-1 locus was detected in 2021-H, 2022-T, and the average environment, and the phenotypic variation rates were >10%, indicating that the PH trait of this locus was less affected by the environment. It was the dominant QTL locus controlling the trait of the plant.

Nine QTLs associated with SD were identified in four linkage groups: LG7, LG8, LG9, and LG10. Four QTLs, namely qSD-8-1, qSD-8-2, qSD-8-3, and qSD-10-1, were detected in at least two environments, explaining the 6–14% phenotypic variation with negative additive effects, indicating a large influence by P2. The other five QTLs were detected in only one environment. Although they were notably affected by the environment, they explained between 12.15% and 25.65% of the phenotypic variation, and were the main effect were associated with SD. These QTLs will be located in future studies.

Four QTLs related to TN traits were identified. These were distributed on LG2, LG7, and LG9, and the phenotypic contribution rate was more than 10%. Only the additive effect of qTN-2-1 was negative, indicating that P2 had a promoting effect on the QTL, while the additive effect of qTN-7-1, qTN-9-1, and qTN-9-2 were positive, mainly promoting the effect of P1. No RFH-QTL was associated with TN, indicating that the environment strongly influenced TN.

For leaf-related traits (LL, LW, and LN), 7, 12 and 9 QTLs were detected for LL, LW, and LN, respectively (**Table 3** and **Figure 4**). These explained 5.96% (AV, qLN-7-4) to 30.97% (2021-H, QLN-7-3) of the phenotypic variation. The additive effect values of LL and LN were mostly negative, and only two and one were positive, indicating that P2 increased the effect value of QTL in LL and LN. On the contrary, LW was considerably affected by P1, which was consistent with the results of the phenotypic traits analysis, indicating that LW was mainly controlled by P1.

Ten QTLs were associated with FW on LG1, LG6, LG9, and LG10, with the highest value of a single QTL being 5.7, explaining 4.9% (AV, qFW-10-3) to 52.44% (AV, qFW-6-2) of the phenotypic variation. All QTLs showed positive additive effects, and P2 increased the QTL effect. The loci qFW-10-1 (167.7–169.3 cM), qFW-10-2 (0.7–3.4 cM), qFW-10-3 (0.7–3.4 cM), and qFW-10-4 (23–26.4 cM) (related to FW) were located on LG10 under three environments and the average environment. However, due to the influence of the environment, the four QTLs were different from the same main locus, and the genetic differences were substantial.

### 3.3 RHF-QTL and QTL enrichment

Fifty-five QTLs associated with sorghum-sudangrass yield traits were identified in three environments and the average environment, which were relatively evenly distributed across 10 LGs. RHF-QTL of qph-7-1, qPH-7-2, qSD-8-1, qSD-8-2, qSD-8-3, qSD-10-1, qLL-10-1, qLW-4-1, qLW-4-3, qLN-7-2, qLN-7-3, qLN-7-4, qFW-1-1, qFW-6-1, qFW- 9-1, qFW-10-3, and qFW-10-4 were repeatedly detected in at least two environments, and no stable QTL association with TN was detected (**Table 4**). Among these RHF-QTL, 6 showed a positive additive effect, and 11 showed a negative additive effect. The phenotypic variation was between 4.9% and 30.97%. qPH-7-1 and qPH-7-2, which control PH, were detected in 2021-H, 2022-T, and the average environment, with LOD values between 3.0–5.2, the highest phenotypic variation explained was 11.66% and 12.45% (>10%). Therefore, it is suggested that qPH-7-1 and qPH-7-2 may be the key QTLs controlling PH. We also found that the loci qLL-10-1 (23–26.6 cM, 25.81 cM) and qFW-10-4 (23–29.6 cM, 25.81 cM), related to the control of LL, overlapped on LG10 with QTL enrichment, which will be further explored in future research. In addition, compared to previous studies, QTL loci related to LW, LN, and FW overlapped or were close to the loci related to previous studies, which further confirmed the mapping accuracy (**Table 5**).

**Table 4:** Relatively high-frequency quantitative trait loci (RHF-QTLs) detected in multiple environments (at least two) statistical analysis.

Traits	QTLs	Treatments	Marker interval	LODs ^a^		Additive effects		R^2^ (%)	
				Max	Min	Max	Min	Max	Min
PH	qPH-7-1	2021-H、2022-T、AV	4.2-5.5	5.2	3.4	16.45	9.8	11.66	9.2
	qPH-7-2	2022-H、2022-T、AV	7-10.5	4.4	3.0	16.73	9.83	12.45	7.76
SD	qSD-8-1	2021-H、AV	48.3-50.5	3.2	4.2	-0.59	-0.75	14	9.7
	qSD-8-2	2021-H、AV	39.9-41.2	3.7	2.6	-0.41	-0.51	13.05	9.5
	qSD-8-3	2021-H、AV	41.2-42.6	3.4	2.6	-0.47	-0.45	11.77	9.04
	qSD-10-1	2022-T、AV	225.4-230.2	3.2	4.7	-0.12	-0.48	6.0	12.19
LL	qLL-10-1	2021-H、AV	23-26.6	4	3.5	-1.41	-2.8	11.61	9.11
LW	qLW-4-1	2021-H、2022-H	145.6-151.8	3	2.8	-0.19	-0.3	9.83	8.00
	qLW-4-3	2022-T、AV	143.2-149.4	3.2	2.9	0.05	-0.07	7.61	8.50
LN	qLN-7-2	2021-H、AV	202-204.1	4.1	2.6	-0.3	-0.43	25.33	16.38
	qLN-7-3	2021-H、AV	204.1-212.4	5.4	2.6	-0.37	-0.66	30.97	12.75
	qLN-7-4	2021-H、AV	94.4-98.4	3.9	2.6	-0.32	-0.58	14.84	5.96
FW	qFW-1-1	2022-T、AV	18.1-20.1	5	3.1	26.48	25.1	16.54	14.61
	qFW-6-1	2022-T、AV	134.9-140.9	3.6	3.1	51.24	20.63	21.39	21.22
	qFW-9-1	2022-T、AV	150.1-151.8	5.6	4.1	3.69	1.58	5.6	5.05
	qFW-10-3	2022-H、AV	7.2-10.4	5.7	2.5	22.15	21.02	22.94	4.9
	qFW-10-4	2022-T、AV	23-26.4	3.4	2.6	-20.11	-41.02	12.75	8.26

a LOD, logarithm of odds.

**Table 5:** Quantitative trait loci (QTLs) mapped on the same chromosome or adjacent marker regions in the current study and previous studies.

Chromosomes	The closest markers	QTLs in this study	QTLs detected in previous studies	
			Related traits ^a^	Reference
4	qlw2	qLW-4-4(AV)	LW	Shi et al. (2017) ^[19]^
5	LN2-1	qLN-5-1(AV)	LN	Yu et al. (2018) ^[21]^
6	QFBMS6.1	qFW-6-1(2022-T,AV)	FW	Wang et al. (2014) ^[39]^
	qFW6			Jin et al. (2021) ^[25]^
	qTW6			Kajiya-K et al. (2020) ^[40]^

a Related traits, LW, leaf width; LN, leaf number; FW, fresh weight.

### 3.4 Candidate gene prediction

A total of 132 annotation genes were screened using gene mining of the RHF-QTL mapping intervals (**Supplementary Table 3**). According to the functional comparison of sorghum homologous genes, seven candidate genes that may affect the yield traits of sorghum-sudangrass were screened. The homologous genes are listed in **Table 6**. Among them, the homologous sorghum gene of *gene23531* was LOC8071161, encoding LRR receptor-like serine/threonine-protein kinase; the homologous gene of *gene26589* in sorghum was LOC8068853, encoding psbP domain-containing protein; the *gene15585* sorghum for LOC8056062 homologous gene, encoding galacturonosyltransferase protein; the sorghum homologous gene of *gene15584* was LOC8059564, encoding magnesium transporter NIPA2 protein; the homologous gene of *gene23381* in sorghum was LOC8080898, encoding DNA polymerase IA, chloroplastic protein; the homologous gene of *gene24523* in sorghum was LOC8054823, encoding RNA polymerase II transcription subunit 15a protein; and the homologous gene of gene31524 in sorghum was LOC8061987, encoding cytochrome P450 711A1 protein.

**Table 6:** Annotated genes in interval of relatively high-frequency quantitative trait loci (RHF-QTLs).

Trait	QTL	Candidate genes	Homologous genesin *Sorghum bicolor* L.	Functional annotation
PH	qPH-7-2	*gene23531*	*LOC8071161*	LRR receptor-like serine/threonine-protein kinase
SD	qSD-8-1	*gene26589*	*LOC8068853*	psbP domain-containing protein
LW	qLW-4-1	*gene15585*	*LOC8056062*	galacturonosyltransferase 8
		*gene15584*	*LOC8059564*	probable magnesium transporter NIPA2
LN	qLN-7-2, qLN-7-3	*gene23381*	*LOC8080898*	DNA polymerase IA, chloroplastic
	qLN-7-3	*gene24523*	*LOC8054823*	RNA polymerase II transcription subunit 15a
FW	qFW-10-3	*gene31524*	*LOC8061987*	cytochrome P450 711A1

## 4 Discussion

The rapid development of molecular biology and the wide application of high-throughput sequencing technology have promoted new breeding strategies to increase crop yield and improve important yield-related traits. Several genetic linkage maps have been created, and some progress has been made in the QTL mapping of related traits. However, most of the existing genetic maps were constructed using restriction fragment length polymorphism (RFLP), sequence-related amplified polymorphism (SRAP), and simple-sequence repeats (SSR), which present few markers and large QTL confidence intervals, limiting their use in QTL fine mapping and marker-assisted breeding (Liu et al., 2015; Shi et al., 2017; Yu et al., 2018; Wang et al., 2021). Compared with the above molecular markers, SNP markers are widely used in map construction and QTL mapping of a variety of crops due to their high density, uniform and extensive distribution on chromosomes, and high genetic stability (Smulders et al., 2019; Jiang et al., 2021; Zhang et al., 2021). Our research group previously constructed a high-density genetic linkage map of the sorghum-sudangrass hybrid, which contained 5656 SNP markers, covering a total genome length of 2192.84 cM, and the average distance between markers was 0.39 cM. (Lu et al., 2022) Compared with the previously constructed maps, the marker density increased, which effectively improved the accuracy of QTL mapping and provided a possibility for further screening of key genes and fine mapping of QTLs.

With the rapid development of sequencing technology and cost reductions, an increasing number of crop genomes have been exploited and applied in related studies (Li et al., 2021). Methods to detect QTL loci physically similar to or overlapping with the reference genome by combining specific crop genome sequences (at the chromosomal level) and high-density genetic maps have been widely used in the rapid identification of QTL loci and potential candidate genes (Luo et al., 2020). Since the sequencing of sudangrass has not been completed, Jin et al. (2021) reported that it is feasible to use the sorghum genome as the reference genome of the sorghum-sudangrass hybrid. In this study, 55 QTLs related to yield traits were detected in three environments based on a high-density genetic linkage map (**Table 2**). Markers located in the same or similar positions on the same chromosome as those in previous studies are listed in **Table 4**. qFW-6-1 is a stable QTL associated with FW and was detected in two of the three environments. In contrast to the previous studies, qFW-6-1 (2402139–50582068 bp) was close to QFBMS6.1 (47686626–50991177 bp) (Wang et al., 2014), qFW6 (45, 156, 899–55, 463, 230 bp) (Jin et al., 2021), and qTW6 (49894350–51216671 bp) (Kajiya-K et al., 2020), which explained 21.22–21.39% of the phenotypic variation, and is an important QTL associated with FW. The marker qLW-4-4 (170.61 cM) was close to the P9m58-453-P9m58-208 marker (171.1 cM) (Shi et al., 2017) (with a negligible difference of 0.49 cM between the two sites), and the phenotypic variation was as high as 25.95%, which was the main effect site of LW. Furthermore, qLN-5-1 is located at 20.41 cM (19.8–22.3 cM) on LG 5, close to marker LN2-1 (22.9 cM) (Yu et al., 2018), with a phenotypic variation of 14.37%. It is a major QTL associated with LN. In addition, most QTLs located on the same LG were detected in the new marker interval due to the differences in marker types, population types, size, and material planting environments used to construct genetic maps (Guo et al., 2020). For PH, two stable QTLs were identified, namely qPH-7-1 and qPH-7-2, which were located at 4.71 cM and 8.11 cM on LG 7. The qPH-7-1 and qPH-7-2 LOD values were 3.4–5.2 and 3.0–4.4, respectively, and the additive effect between alleles was positive, which explained 7.76–11.66% of the phenotypic variation, indicating a stable QTL locus associated with PH. Compared with previous studies, qPH-7-1 (4.2–5.4 cM) and qPH-7-2 (7–10.5 cM) were co-localized with qPH7 (110.3–112.92 cM) loci on chromosome 7 (Zou et al., 2012). However, both qPH-7-1 and qPH-7-2 were inconsistent with qPH7 markers, suggesting that these may be novel QTLs for PH. In subsequent studies, these QTLs will be the focus of our attention. Additionally, compared with previous studies, it was found that several QTLs related to yield traits were detected at different positions on the same chromosome. For example, the QTLs controlling SD was detected on LG7, LG8, LG9, and LG10 (Wang et al., 2014; Liu et al., 2015; Kong et al., 2020; Jin et al., 2021); that controlling LL on LG3, LG4, LG7, LG8, LG9, and LG10 (Wang et al., 2014; Shi et al., 2017; Yu et al., 2018); and the one related to TN on LG2 (Li et al., 2015; Rayaprolu et al., 2021).

Yield traits such as PH, SD, TN, leaf-related traits, and FW are important quantitative traits, which are influenced considerably by the environment. The results of QTL localization are different in different environments, and the accuracy of the localization results can be guaranteed by setting up multi-year and multi-environmental tests to verify the QTL detected in multiple environments (Wang et al., 2009; Hou et al., 2015). Generally, QTLs that can be located in multiple environments (at least two environments) or all environments and have similar effects are defined as stable QTLs; otherwise, they are considered to be greatly influenced by the environments that they interact with (Xie et al., 2008). Guo et al. (2020) detected 38 QTLs related to protein and 68 related to starch in three environments and the average environment, respectively, and 26 stable QTLs were detected in more than two environments. Liu et al. (2022) identified 183 QTLs related to cotton fiber and yield traits in six environments, 62 QTLs for fiber and 10 QTLs for yield stability were identified in multiple environments. Ma et al. (2018) mapped maize leaf-related traits in three environments and eight stable QTLs in two or three environments, explaining 4.38–19.99% of the phenotypic variation. Additionally, Yang et al. (2022) identified 105 QTLs related to cotton in three environments, a total of 25 stable QTLs were detected in more than two environments. In this study, 55 QTLs related to yield traits of the sorghum-sudangrass hybrid were mapped in 3 environments and the average environment, and 17 RHF-QTLs were repeatedly detected in at least two environments (**Table 3**). Among them, there were two stable sites related to PH, four in SD, one in LL, two in LW, three in LN, and five in FW. These could explain 4.9–30.97% of the phenotypic variation. qPH-7-1 and qPH-7-2, which control plant height, could be detected in 2021-H, 2022-T, and the average environments, explaining 7.76–13.56% of the phenotypic variation (>10%), which may be stable QTL controlling PH formation. Meanwhile, we also found that a pair of stable QTLs controlling different traits were co-localized at the same position and chromosome. The qLL-10-1 (23–26.6 cM, 25.81 cM) and qFW-10-4 (23–29.6 cM, 25.81 cM) markers were completely or only partially overlapped in LG10.

The LRR receptor-like serine/threonine-protein kinase encoded by the *gene23531* has high homology with the brassinolide insensitivity (BRI) gene and jointly regulates brassinosteroid signals. Ou et al. (2015) investigated dwarfing and non-dwarfing rootstock pear varieties based on RNA-sequence, and the results showed that the LRR receptor-like serine/threonine-protein kinase, a key gene controlling PH growth, was significantly up-regulated in the dwarfing varieties (Li et al., 2002). The psbP domain-containing protein encoded by *gene26589* participates in plant photosynthesis and plays an important role in the assembly of plant PS II and maintaining conformation stability (Bricker et al., 2013). Many studies have shown that the suppression of PsbP will cause the decline of the oxygen evolution ability of plants, a change in the direction of the electron transfer chain, and a lack of PS II oxidation and reduction function (Yi et al., 2007; Ido et al., 2009). The *gene15585*, coding the galacturonosyltransferase 8 (GT8) family proteins, is divided into two branches. The former mainly includes subclades of galacturonosyltransferase (GAUT) and galacturonosyltransferase-like TL (GA) genes, which play an important role in the synthesis of cell walls (Cheng et al., 2018). Kong et al. (2011) found that GAUT1 was involved in the synthesis process of pectin. In a tomato study, de Godoy et al. (2013) found that GAUT4 gene silencing treatment significantly reduced the pectin content. Therefore, it was speculated that *gene15585* might be key in leaf growth. The magnesium transporter NIPA2, encoded by the *gene15584*, is involved in plant photosynthesis and plays an important role in leaf growth and aging delay. Hermans and Verbruggen (2005) and Horlitz and Klaff (2000) also found that magnesium transporter NIPA2 is an important Mg^2+^ transporter, which could accelerate the continuous transport of Mg^2+^ to green tissues such as leaves, promoting the synthesis of green pigment and carbon assimilation and accelerating plant growth. It was speculated that the gene might be related to leaf photosynthesis. Chloroplasts are semi-autonomous organelles that contain their own DNA and can self-replicate. The *gene23381* and *gene24523* encode chloroplast DNA polymerase and RNA polymerase, respectively, which are key enzymes in plant regulation of chloroplast DNA synthesis and transcription in plants. RNA polymerase participates in the synthesis of various mRNA species, transcribes tRNA genes, regulates rRNA synthesis, and plays an important role in maintaining the growth and development of plant leaves (Börner et al., 2015). Zoschke et al. (2007) measured chloroplasts in *Arabidopsis* seeds and young, and old leaves at the transcriptional level and found that the transcriptional activity was relatively stable in all three. In this study, *gene23381* and *gene24523* were annotated in qLN-5-1, a QTL related to LN traits, suggesting that these genes may be important in controlling the growth and development of leaves of the sorghum-sudangrass hybrid. The *gene31524* encodes plant cytochrome P450. Renault et al. (2014) found that it has high catalytic activity, participates in a variety of metabolic reactions in plants, and plays an important role in signal transduction, pigment synthesis, light, electron transport, and biological defense. Therefore, it is speculated that *gene31524* may be the key gene affecting the growth and metabolism of the sorghum-sudangrass hybrid.

Under different environmental conditions, QTL loci detected at the same or adjacent loci on the same chromosome are called “QTL hotspots” or “QTL clusters,” which are the result of the regionalized distribution of QTLs related to different traits (Yang et al., 2022). They are also the preferred regions for fine mapping and candidate gene identification (Xie et al., 2008). It can introduce genes related to crop quality, yield, and resistance into recipient crops simultaneously, control the correlation between traits in stable “QTL hotspots” or “QTL clusters” of different traits, and regulate pleiotropy of various traits through different metabolic pathways (Huang and Yan, 2019; Waheed et al., 2021). Numerous studies have shown that the phenomenon of “QTL hotspots” or “QTL clusters” are prevalent in a variety of crops, such as crested wheatgrass (Yang et al., 2022), wheat (Cui et al., 2016), and sorghum (Mace et al., 2012). In this study, we found that qLL-10-1 and qFW-10-4 were located at the same position of 25.81 cM on LG10 among the seven stable QTL controlling yield traits of sorghum-sudangrass hybrid. In addition, FW was significantly positively correlated with LL, in agreement with previous studies.

## 5 Conclusions

In this study, 55 major QTLs related to PH, SD, TN, LL, LW, LN, and FW were identified based on the high-density SNP map of the sorghum-sudangrass hybrid, among which 17 relatively RHF-QTL were detected in at least two environments. A stable QTL cluster containing QTLs controlling LL and FW (including at least one RHF-QTL) was detected, and three QTLs overlapping or located adjacent to the previously studied sites were identified. The genes in the RHF-QTL intervals were annotated, and seven candidate genes that might be related to PH, SD, LW, LN, and FW were screened. The results of this study will promote the fine mapping of QTL for yield traits of sorghum-sudangrass hybrids, cloning of key genes, and marker-assisted breeding.

## Data availability statement

The data presented in the study are deposited in the figshare repository, accession number https://doi.org/10.6084/m9.figshare.21717146.v1.

## Author contributions

ZY and QL conceived and designed the study. ZY, XY and QL performed the experiments. QL and XY wrote the article. HW, XZ, and YZ assisted in the performance of the experiments. ZY is the corresponding authors at the request of the Institute. All authors have read and approved the final manuscript.

## Supplementary material

Supplementary Table 4 Phenotype data of Parental. 10.6084/m9.figshare.21667115
